# Prevalence of poor self-rated health and associated risk factors among older adults in Cali, Colombia

**Published:** 2013-12-31

**Authors:** José M Ocampo-Chaparro, Helmer de J Zapata-Ossa, Ángela M Cubides-Munévar, Carmen L Curcio, Juan de D Villegas, Carlos A Reyes-Ortiz

**Affiliations:** 1 Departamento de Medicina Familiar, Universidad del Valle, Cali, Colombia jmocampo2000@yahoo.com.ar; 2 Escuela de Medicina, Fundación Universitaria San Martín, Grupo de Investigación en Salud Pública (GISAP), Cali, Colombia. zapatahelmer@gmail.com; 3 Departamento de Accion Física Humana, Universidad de Caldas, Manizales, Colombia. carmen.curcio@ucaldas.edu.co; 4 Escuela de Medicina, Fundación Universitaria San Martín, Cali, Colombia. sanmartin.coordinacionclinica@gmail.com; 5 Gerontology Institute, Georgia State University, Atlanta, GA, USA. careyesortiz@hotmail.com

**Keywords:** Self-rated health, older adults, aging, frailty, biopsychosocial model, Colombia

## Abstract

**Introduction:**

Self-rated health (SRH) has beeen considered an important marker of quality of life and an independent predictor of mortality in older adults.

**Objective:**

To determine the prevalence of poor SRH and identify risk factors associated with poor SRH among older adults residing in the Commune 18 of the city of Cali, Colombia, in 2009.

**Methods:**

A population-based cross-sectional study with a single-stage cluster sampling design. Sample included 314 persons aged 60 and older. The dependent variable, SRH was dichotomized into good (excellent, very good, good) and poor (fair, poor). Independent variables were sociodemographic, biological, mental, functional and geriatric syndromes. Logistic regression was used for multivariate statistical modeling.

**Results:**

Overall, 40.1% reported poor SRH (women 42.9%, men 35.0%). Factors independently associated with poor SRH were diabetes mellitus, depression, fear of falling and frailty syndrome (frail and pre-frail vs. non-frail). Widowed men reported poorer health than married men while other marital status (single/separated/divorced) was associated with better self-rated health in women.

**Conclusion:**

Potential modifiable factors such as depression and frailty syndrome are important determinants for poor SRH in Colombian older adults.

## Abstract (es)

**Introducción:**

El auto-reporte de salud (SRH) se ha considerado un marcador importante de calidad de vida y un factor predictor independiente de mortalidad en adultos mayores.

**Objectivo:**

Determinar la prevalencia de SRH pobre e identificar los factores de riesgo asociados con ARS pobre en adultos mayores que viven en la Comuna 18 de la ciudad de Cali, Colombia, en 2009.

**Métodos:**

Un estudio poblacional de corte transversal con un diseño de muestreo por conglomerado monoetápico. La muestra incluyó 314 personas de 60 años o más. La variable dependiente, SRH fue dicotomizada en buena (excelente, muy buena y buena) y pobre (regular or mala). Las variables independientes fueron sociodemográficas, biológicas, mentales, funcionales y síndromes geriátricos. La regresión logística fue usada para modelos estadísticos multivariados.

**Resultados:**

En general, 40.1% reportaron SRH pobre (mujeres 42.9%, hombres 35.0%). Los factores asociados independientemente con SRH pobre fueron diabetes mellitus, depresión, temor a caer y síndrome de fragilidad (frágil and pre-frágil vs. no-frágil). Los hombres viudos reportaron más SRH pobre comparados con hombres casados, mientras teniendo otro estado marital (soltero, separado, divorciado) fue asociado con menos SRH pobre en mujeres.

**Conclusión:**

Factores potencialmente modificables tales como la depresión y el síndrome de fragilidad son determinantes importantes de una pobre SRH en adultos mayores colombianos.

## Introduction

The aging of the population is the main demographic phenomenon in the world in the late twentieth and early twenty-first century. Indeed, the older adult population, defined as those aged 60 or more years by the World Health Organization, presents growth rates of around 2.4%, compared with 1.7% of the general population^1^. Colombia is no stranger to demographic change that occurs worldwide. This is reflected in its population of over 40 million people and the rapid growth in the number of older adults, which went from 600,000 in 1950 to 3,000,000 in 2001, and is projected to 14,400,000 in the year 2050^2^. Aging may lead to a gradual deterioration of physical health conditions, mental and social activities of the individual, and as result, the functional status may be affected by limitations of activities of daily living. Similarly, health status changes that occur with aging are more chronic than acute and more progressive than regressive. This requires the assessment of the general health needs of this population for planning of health promotion and disease prevention as well as development of healthcare services^3^.

It is noteworthy that the concept of health integrates both objective and subjective components; a way of evaluating the subjective component is through assessing the self-rated health (SRH),that is a person's perception of their actual health^4^. SRH integrates information on the different dimensions of the individual: biological, mental, social, functional status and the presence of geriatric syndromes^5^. It is therefore considered to represent the perception of the individual as a whole, on different dimensions of their health status, and hence may be classified as a multidimensional variable outcome^6^. Indeed, SRH has been considered an important dimension of quality of life worthy to be investigated in older adults^5^. In addition, SRH has been found an important marker and independent predictor of mortality in older adults^6^ ^,^ ^7^. 

In Colombia there has been little research on SRH and particularly in the older adult population. One of these was developed by Gómez *et al*.,^8^ based on a cross-sectional study in older adults of the city of Manizales, where they explored the SRH and its correlations with the presence of comorbidity and functional status. However, SRH includes other domains that were not evaluated in their study. Based on our multidimensional conceptualization, we wanted to identify variations by gender on social, biological, mental and functional factors and geriatric syndromes that are correlated with poor SRH in the older adult population. Our objective in this study was to determine the prevalence of poor SRH and identify risk factors associated with poor SRH among older adults residing in the Commune 18 of the city of Cali, Colombia, in 2009.

## Material and Methods

### Design and sample

We performed a population-based cross-sectional study in 2009, in community-dwelling older adults residing in Commune 18, an underserved urban area of the city of Cali, Colombia. The study population included older adults representing 8% of the total population in that area (97 707 people in 2008)^9^. Inclusion criteria were being 60 years or older, a resident of the Commune 18 for a period greater than or equal to six months, agreeing to participate in the study and sign an informed consent. Exclusion criteria were residing in nursing homes, because it is known to have a higher probability of a worse state of deterioration in health or functional status, or having severe hearing loss, because it prevented the accuracy of the assessment.

The sample was obtained through a single-stage cluster sampling in which the block was considered as the sampling unit. The sampling frame corresponded to the database of 387 records supplied by the Secretariatof Planning of the municipality of the city of Cali for the year 2006, belonging to socioeconomic strata one, two and three^9^. The number of sampling units or clusters corresponded to 30 blocks formed on average for 25 homes. It was considered one older adult for every 2.25 households, 1.1 households per house, 25 houses per block and a total of 30 blocks to be interviewed. To calculate the sample, consideration was given to the following factors: a non-response factor of about 15%, a reliability of 95% (Z1-α/2= 1.96), an estimated error of approximately 7.2%, an expected prevalence of 50 % for poor SRH (in order to maximize the sample size p=0.5 q=1-p= 0.5from a total number of 7,376 older adults residing in the Commune 18), and an adjustment of the design effect of two. The detailed description of sampling design for the research was published elsewhere^9^. For a planned sample size of 364, the final sample in this study was composed of 314 participants, with a response rate of 86.3%. 

The data collection was performed with interviewers who were previously trained in health education by the principal investigators. A pilot test was performed to check the clarity of the survey's questionnaire and adjust the tools and skills of field staff. Field supervisors verified the quality of information provided by the older adults. Face-to-face interviews and physical measures were then performed. Responses to the questionnaire and measures were collected and saved using the EpiInfo 2002. The study was approved by the ethics committee of the University Foundation St. Martin (FUSM) and the Universidad del Valle.

### Measures

### Dependent variable

Self-rated health was considered as the outcome. It was assessed by this question: "In general would you describe your health as". There were five possible answers: "excellent", "very good", "good", "fair" or "poor". Good SRH was defined by any of the first three answers, and poor SRH by any of the last two. 

### Independent variables

The independent variables were grouped into five subgroups: sociodemographic, biological, mental, functional and geriatric syndromes**. **Sociodemographic variables were age, marital status, socioeconomic status, literacy, education level, occupation, and health insurance affiliation. Age (years) included these groups: 60-69, 70-79, and 80+. Marital status included married, widowed and other. Socioeconomic status included 1= low-low, 2= low and 3= medium-low; these categories reflect the government area-based socioeconomic stratification based on physical characteristics of the houses, surrounding environment, and urbanistic context. Literacy included being able to read and write (yes/no). Education categories included none, primary, and secondary (high school) or technical/university. Occupation categories included worker, homemaking, retired, and unemployed/other. Health insurance affiliation included these categories: contributory (worker pay to insurer), subsidized (government pay to insurer), and non-affiliate (none). 

Biological factors were medical conditions and body mass index (BMI). Medical conditions were assessed by asking participants if they had been told by a physician that they had arthritis, cardiac disease (heart failure or ischemic heart disease), diabetes, or hypertension. Staff field who were trained by the principal investigators made the anthropometric measurements. BMI was computed by dividing weight in kilograms (kg) by height in meters square. Metric wall rules and counterweight balance scales were used to measure height and weight. 

Mental variables were cognitive function and depression. Cognitive function was assessed with the Spanish validated version of the Mini-Mental State Examination (MMSE) scale^10^. This test assesses the cognitive domains of memory, orientation, attention, language, visual-constructional function and praxis. The MMSE has a maximum score of 30, with higher scores representing better performance. It has a sensitivity of 92.3% and specificity of 53.7% in the Colombian population^11^. Since it is highly correlated to education level among Latinos, to indicate cognitive impairment we chose the cut-offs that are recommended in the Colombian older population: 1- <18 for illiterate, 2- <21 for primary education, and 3- <24 for secondary education or higher^12^. The presence of depression was assessed using the Yesavage Geriatric Depression Scale (GDS-15), which has a sensitivity between 80 to 90% and specificity between 70 and 80% for a cut-off ≥6^13^, and the Spanish version was used that has been validated in the older Colombian population^14^. The score ranges from 0 to 15 points; it was classified as 0-5 normal, 6-10 moderate depression, and 11-15 severe depression. The cut-off value of ≥6 was chosen to indicate clinically important depressive symptoms^13^.

Functional status was evaluated by eight instrumental activities of daily living (IADL) of a Colombian validated version in Spanish^15^ of the Lawton and Brody scale and 10 basic activities of daily living (ADL) of the Spanish validated version^16^ of the Barthel index. The Lawton and Brody scale included the ability to use the telephone, shopping, food preparation, housekeeping, laundry, mode of transportation, responsibility for own medications, and ability to handle finances. The person receives a score of 1 for each item that is considered independent and 0 for each item that is considered dependent; the total score may range form 0-8, where a lower score indicates a higher level of dependence. One or more with zero responses was considered a person with dependence in the Lawton and Brody scale. The Barthel index included feeding, bathing, grooming, dressing, bowels continence, bladder continence, toilet use, transfers (bed to chair and back), mobility (on level surfaces), and climbing stairs; the final score ranges from 0 to 100, where 0 is maximum dependence and 100 is total independence. Lower scores indicate greater dependence. The Barthel index was categorized as <80 (dependent) and 80-100 (independent). 

Geriatric syndromes were polypharmacy, falls, fear of falling, and frailty. Polypharmacy was defined as currently taken three or more medications. Falls were assessed by the following question: "During the past year, how many times did you fall and land on the floor or ground?". Falls was dichotomized as no falls vs. one or more falls. Fear of falling was evaluated by this question: "Are you afraid of falling" Yes or no. 

### Frailty

The assessment of frailty syndrome was performed based and adapted from the phenotype described by Fried and colleagues^17^ which includes five criteria: shrinking, weakness, exhaustion, slow walking speed and low level of physical activity (Table 1). A person having any of the criteria was assigned a score of 1, with a potential summative score range from 0 to 5. Frail older adults were considered those who met three or more criteria, pre-frail those who met one or two of these criteria and non-frail those who did not have any of them. All five components from the original phenotype were retained fro our study; however, two of the measures were slightly different (exhaustion and low physical activity) and defined as follows:

**Table 1:**
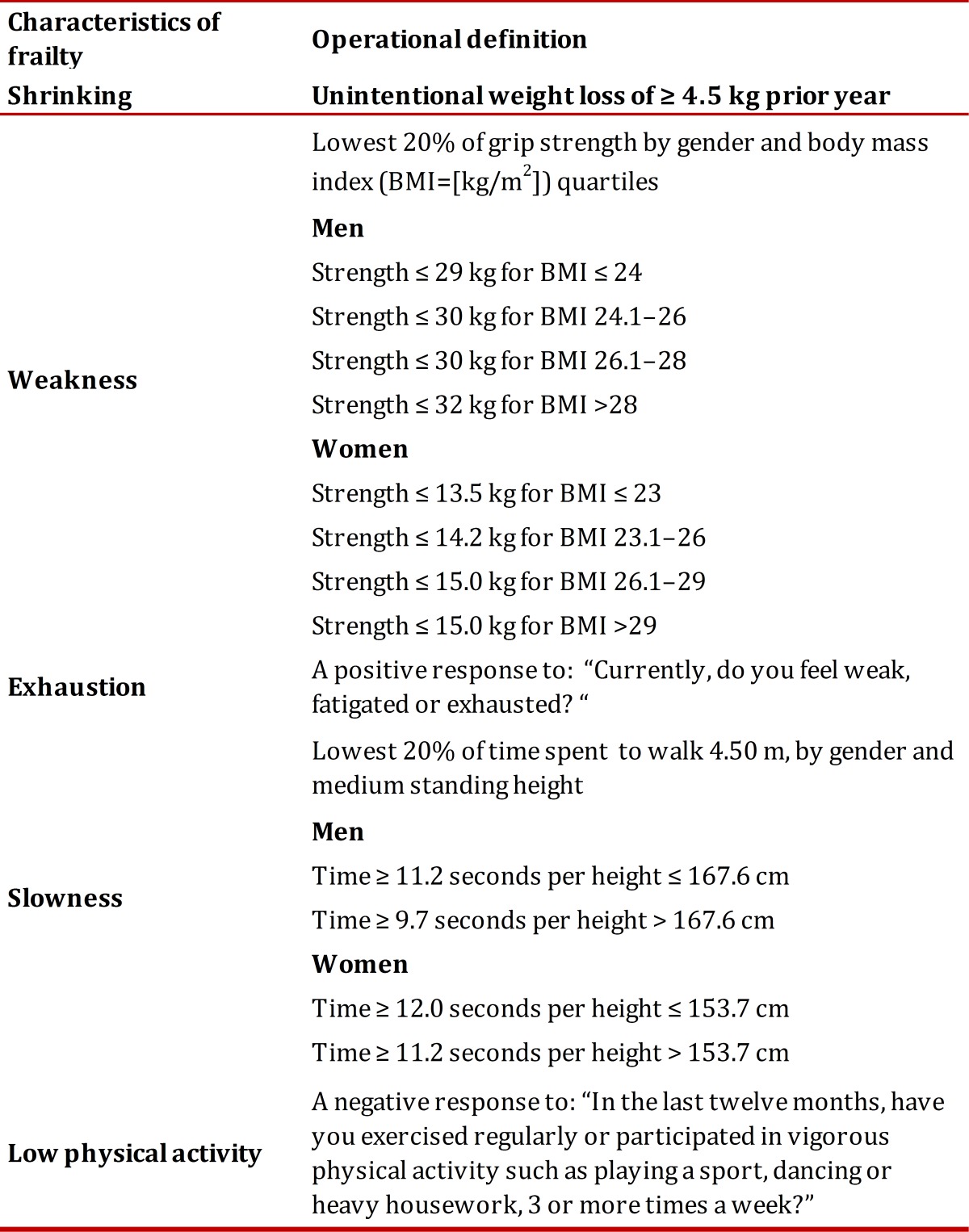
Description of frailty

### Shrinking

Weight loss was assessed by this question: "Have you lost weight unintentionally in the prior year". If yes, how many kilograms. If reported 4.5 kg or more, it was considered having weight loss (score= 1)^17^. 

### Weakness

Weakness, or the lowest grip strength, was defined as the lowest 20% hand grip strength (score= 1) stratified by gender and BMI quartiles^17^. Hand grip strength (kg) was measured using an Amedley Hand Dynamometer III (Takei, Tokyo, Japan). Staff field who were trained by the principal investigators made the grip strength measurements. The older person was asked to remain standing with an outstretched arm adducted across the body and shoulder in medial rotation, using the dominant hand with an angle of 90° in the second phalanx. Participants then were instructed to squeeze the handgrip as hard as they could. 

### Exhaustion

 Exhaustion or fatigue was assessed by self-report (yes, score= 1) (Table 1). The original measure used by Fried *et al*.^17^ was based in two questions of the CES-D (Center for Epidemiological Studies Depression) scale; however, in our study we have another depression scale (GDS-15) and we did not wanted an overlap between frailty syndrome and depression that is a very important variable usually correlated to SRH. 

### Slowness

 Persons were asked to walk a 6 m distance at normal walking speed. A digital chronometer was used to measure the time spent during the last 4.50 m. Walking speed was calculated diving the 4.50 m by the time in seconds. Slowness in walking speed was defined as the lowest 20% of walking speed (score= 1), by gender and medium standing height^17^.

### Low physical activity

 Physical activity, was assessed asking for regular exercise or vigorous physical activity, where a negative response was considered a low physical activity level (no, score= 1) (Table 1). We used the same question as in the SABE Study measuring physical activity in Latin American elders^18^. 

### Statistical analyses

**Figure 1:**
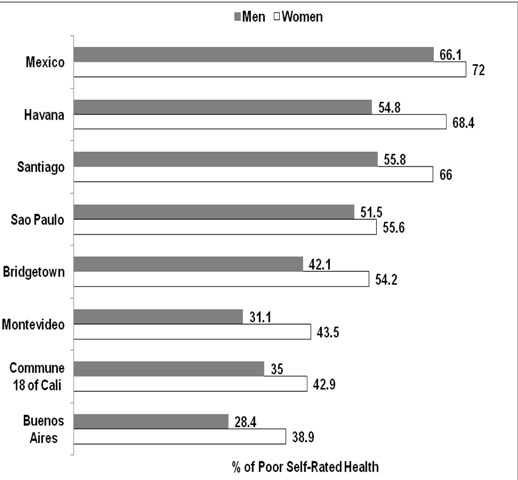
Prevalence of Poor Self-Rated Health among Men and Women 60 Years and Older in Commune 18 of Cali (2009) and other Cities in Latin America and the Caribbean (1999-2000)

To describe differences on SRH (original five responses or dichotomized) by gender (Table 2), proportions and 95% confidence intervals were estimated using the exact method for the binomial distribution. To test differences on poor SRH percentages across the categories of variables in the total sample or separated by gender, we used the Chi-square or the Fisher test (Table 3). The statistical multivariate modeling process was performed using logistic regression (Table 4). The initial analysis was made using the total sample and later separated by gender. Odds ratios (OR) and 95% confidence intervals (CI) were calculated. The fit of the multivariate models was established by using the Hosmer and Lemeshow goodness of fit test. In addition, we calculated weighted and age-adjusted prevalence proportions by the direct method, with the 2000 U.S. population as the standard, and compared Commune 18 of Cali with cities from the Health, Well-Being and Aging in Latin America and the Caribbean Study (SABE) (Fig. 1)^18^ . All statistical analyses were carried out using version 9.2 of Statistical Analysis System (SAS) for windows (SAS Institute: Cary, North Carolina), significance level was considered a *p*<0.05.

## Results

The prevalence of poor self-rated health in the total population was 40.1% (95% CI 32.2-48) and after stratification by gender was 42.9% (95% CI 34.4-51.5) for women and 35.0% (95% CI 23.2-46.7) for men (Table 2). There was no statistical difference between men and women.

**Table 2:**
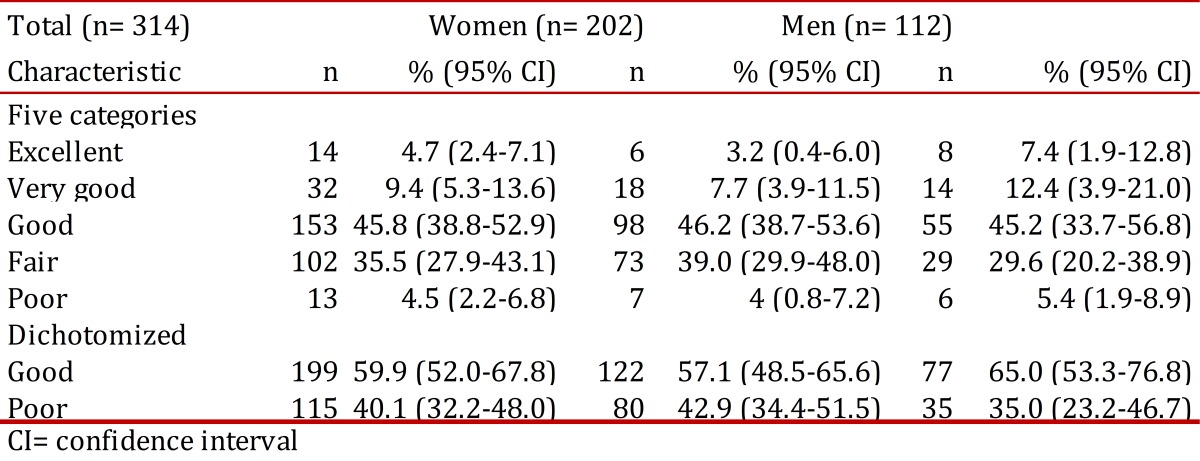
Distribution of prevalence of self-rated health by gender.

Table 3 shows the distribution of poor self-rated health by socio-demographics, biological, functional, and mental variables, and geriatric syndromes. In the total population, variables significantly associated with poor SRH were lower socioeconomic status, lower education level, cardiac disease, diabetes, arthritis, dependence in IADL (Lawton) and ADL (Barthel), higher depression scores, polypharmacy, falls, fear of falling, weakness, exhaustion, slowness in walking, low physical activity and the frailty syndrome. In women, variables significantly associated with poor SRH were lower socioeconomic status, lower education level, cardiac disease, diabetes, arthritis, dependence in IADL (Lawton), higher depression scores, fear of falling, weakness, exhaustion, low physical activity and the frailty syndrome. In men, variables significantly associated with poor SRH were cardiac disease, higher depression scores, falls, exhaustion, slowness in walking, and the frailty syndrome.

**Table 3:**
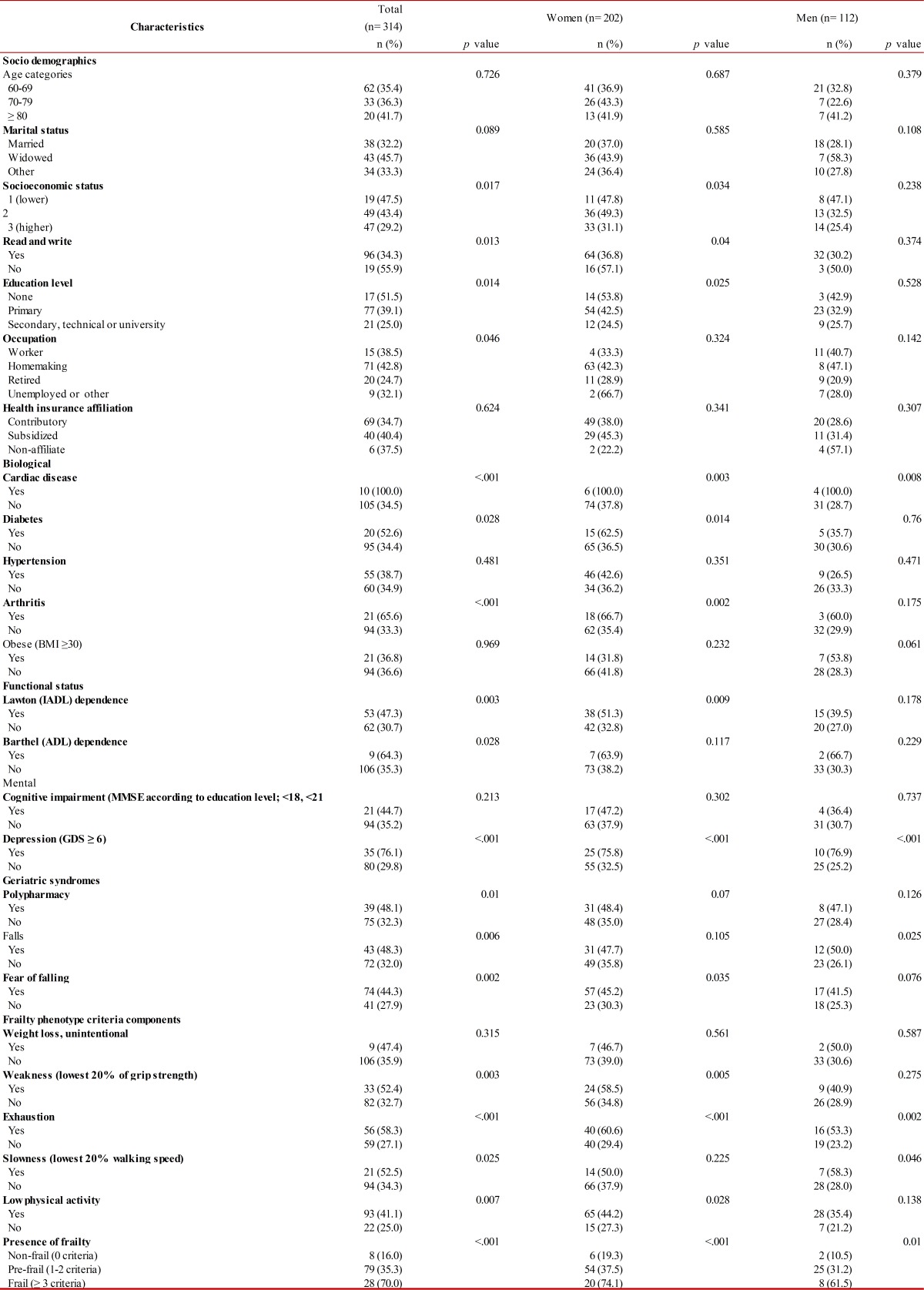
Distribution of poor self-rated health by socio-demographics, biological, functional, and mental variables, and geriatric syndromes.

Table 4 shows the multivariate logistic regression analyses for poor self-rated health as a function of selected independent variables, in the total sample and by gender. Women with other marital status were less likely to have poor SRH, compared to married persons; while widower men were more likely to have poor SRH. In both men and women, depression and fear of falling were significantly associated with poor SRH. Having diabetes was associated with poor SRH in women but not in men. In the total sample, frail participants (three or more criteria of the frailty phenotype) exhibit the highest odds for poor SRH, with about eight times the odds than that non-frail (OR 8.15, 95% CI 3.20-20.77). 

**Table 4:**
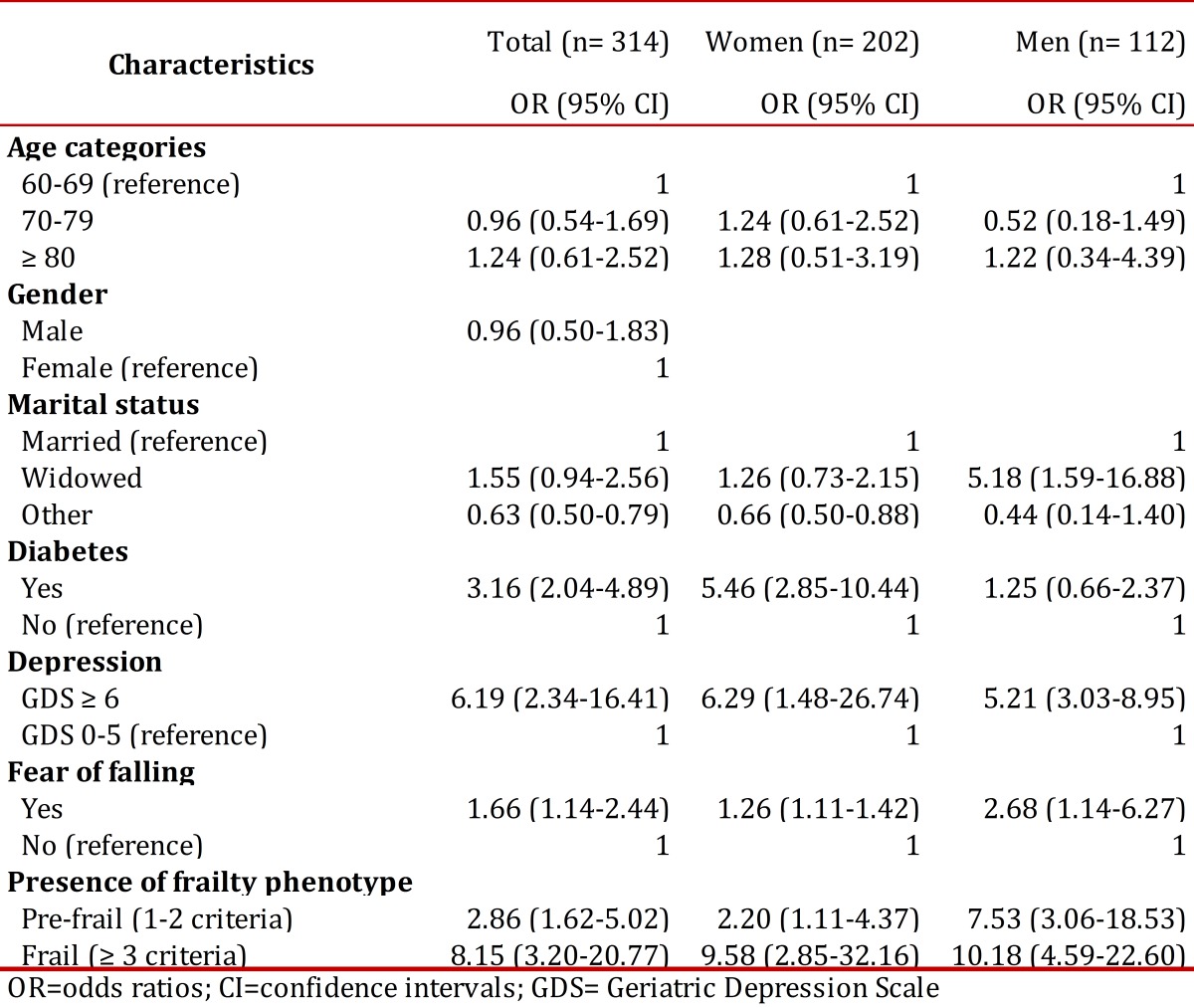
Multivariate logistic regression analyses for poor self-rated health as a function of independent variables, pooled sample and by gender.

Figure 1 shows the prevalence of poor self-rated health by gender in Commune 18 of Cali and across other cities in Latin America. Commune 18 of Cali had lower prevalence of poor SRH in both older men and women than those in Bridgetown, Sao Paulo, Santiago, Havana and Mexico City, but higher prevalence than that in Buenos Aires (men and women) and Montevideo (men).

## Discussion

In this study, the prevalence of poor SRH and associated risk factors were investigated, among community-dwelling older adults residing in the Commune 18 of the city of Cali, Colombia. The prevalence of poor SRH is lower compared to other cities in Latin America. Factors independently associated with poor SRH were diabetes mellitus, depression, fear of falling and frailty syndrome (frail and pre-frail vs. non-frail). 

The age-adjusted prevalence of poor SRH in this study was lower than that reported in most Latin American cities. Although older men had lower SRH than older women in our study, it did not reach statistical significance. In other studies, it is also reported that the proportion of poor SRH is higher in older women than in older men^19^ ^,^ ^20^. The lack of significant difference between men and women on poor SRH in our study is consistent with several studies. In Spanish older people living at home, gender was not associated with poor SRH^19^. In a Brazilian study, gender was not associated with the prevalence of poor SRH among the elderly^21^. The similarities for the self-perceptions of general health in these Ibero-American older populations may indicate that health prevention strategies related to factors affecting SRH should target both older men and women. 

Our findings related to the association between frailty and poor SRH is consistent with another study although with different definitions and measures for frailty, and where SRH was not the main variable of interest. Using data from The Three-City Study, Avila-Fúnes*et al*.^22^, found higher prevalence of poor SRH for pre-frail or frail older individuals, compared to those non-frail. In that study, although their definition for frailty was based on Fried *et al*.^17^, criteria as our study, they used two components of the syndrome in a different way: shrinking (recent weight loss of ≥3 kg) and weakness (no grip strength but a question asking for difficulty rising from a chair). In agreement to these findings, frailty arises as a key factor affecting SRH in older adults. Also, frailty syndrome is an important concern in public health policy because as the aging population increases, more frail older adults will live in the community and they will need particular preventive strategies and health services support. 

Depression was also independently and strongly associated with poor SRH. In a Spanish study^20^ reported an independent association between depressive symptoms and poor SHR among men and women aged 65 years and older. A potential mechanism of the association between depression and poor SRH is that depression may be in the pathway or inter-related to poor SRH. For example, older adults with depression may perceive their general health worse than those without depression. Depression is the most common mental health problem affecting older adults^20^. Then, public health policies related to early detection and prevention of depression, by controlling its risk factors, among older adults in the community are needed. 

We also showed that fear of falling was independently associated with poor SRH, and this is consistent with other studies. In another study, restriction of activities related to fear of falling has been associated with poor SRH^22^. Since fear of falling with or without activity restriction is usually associated with falls, it has also public health policy implications. Indeed, fear of falling may increase the risk for falls among older adults and it is considered the main cause of un-intentional injury in older adults living in the community^22^. 

Diabetes was independently associated with poor SRH in women but not in men. Other studies did not distinguish gender differences in this association. In an analysis using data from the SABE study, Wong *et al*.^23^, found an association between poor SRH and diabetes among Latin American older individuals. Another study has shown an association between poor SRH and diabetes or other metabolic problems^24^. The increased prevalence of diabetes among older adults is also a public health policy issue that should be addressed^24^. Identifying and controlling risk factors (e.g., sugar intake, lack of exercise) for an early detection and management of diabetes is an imperative. 

This study is not without limitations. First, it was conducted in older adults living in the community, in a localized area and not the entire city of Cali, which is a population group with particular social and economic characteristics (e.g., low socioeconomic status community) and did not consider the institutionalized population , rural, or health institutions, potentially limiting the external validity of some of the findings reached. Second, because this was a cross-sectional study, it was not possible to determine the tempo

This study has also some strength and significance. First, it was based in a multidimensional theoretical model and multiple factors associated with poor SRH were identified. These included biological and psychological determinants, and geriatric syndromes such as fear of falling and frailty. Second, it was one of the first population-based studies for SRH among urban older people in Colombia. 

Thus, our findings could be used to assess health status and its determinants in the older population in other areas at the city of Cali and other groups of older adults with similar characteristics (e.g., underserved areas) in Colombia. Our findings can help to provide information for health practitioners and policy makers in developing and implementing programs of health promotion and disease prevention as well as the adequacy and planning of different levels of care for the older populations. These insights will help to respond better to the challenges that the health system faces with greater intensity in the following years given the phenomenon of demographic and epidemiological transition in Colombia and other countries in Latin America^23^. 

## Conclusion

The prevalence of poor SRH in the Commune 18 of the city of Cali is lower compared to other cities in Latin America. Factors independently associated with poor SRH were diabetes mellitus, depression, fear of falling and frailty syndrome (frail and pre-frail vs. non-frail). Thus, potential modifiable factors such as depression and frailty syndrome are important determinants for poor SRH in Colombian older adults. 
